# A Cell-Penetrating Peptide That Blocks Toll-Like Receptor Signaling Protects Kidneys against Ischemia-Reperfusion Injury

**DOI:** 10.3390/ijms22041627

**Published:** 2021-02-05

**Authors:** Su Woong Jung, Jung-Woo Seo, Seon Hwa Park, Yang Gyun Kim, Ju-Young Moon, Sangdun Choi, Sang-Ho Lee

**Affiliations:** 1 Division of Nephrology, Department of Internal Medicine, Kyung Hee University Hospital at Gangdong, Seoul 05278, Korea; ha-ppy@daum.net (S.W.J.); jw11s@hanmail.net (J.-W.S.); 01love14@hanmail.net (S.H.P.); apple8840@hanmail.net (Y.G.K.); kidmjy@hanmail.net (J.-Y.M.); 2 Department of Core Research Laboratory, Medical Science Institute, Kyung Hee University Hospital at Gangdong, Seoul 05278, Korea; 3 Department of Molecular Science and Technology, Ajou University, Suwon 16499, Korea; sangdunchoi@ajou.ac.kr

**Keywords:** kidney, ischemia-reperfusion injury, Toll-like receptors, TLR-inhibitory peptide 1

## Abstract

Renal ischemia-reperfusion injury (IRI) is involved in the majority of clinical conditions that manifest as renal function deterioration; however, specific treatment for this type of injury is still far from clinical use. Since Toll-like receptor (TLR)-mediated signaling is a key mediator of IRI, we examined the effect of a multiple-TLR-blocking peptide named TLR-inhibitory peptide 1 (TIP1), which exerts the strongest action on TLR4, on renal IRI. We subjected C57BL/6 mice to 23 min of renal pedicle clamping preceded by intraperitoneal injection with a vehicle or TIP1. Sham control mice underwent flank incision only. Mouse kidneys were harvested after 24 h of reperfusion for histology, western blot, RT-PCR, and flow cytometry analysis. Pretreatment with TIP1 lowered the magnitude of elevated plasma creatinine levels and attenuated tubular injury. TIP1 treatment also reduced mRNA expression of inflammatory cytokines and decreased apoptotic cells and oxidative stress in post-ischemic kidneys. In kidneys pretreated with TIP1, the infiltration of macrophages and T helper 17 cells was less abundant than those in the IRI only group. These results suggest that TIP1 has a potential beneficial effect in attenuating the degree of kidney damage induced by IRI.

## 1. Introduction

Ischemia-reperfusion injury (IRI) clinically manifests as acute kidney injury following shock, sepsis, or renal transplantation. Under temporary cessation of blood supply, tubular epithelial cells are deprived of oxygen and nutrients and fall into an ATP-deficient state. The subsequent recovery of blood supply accelerates tissue injury by generating reactive oxygen species (ROS). Under these conditions, tubular epithelial cells undergo apoptosis and necrosis and release endogenous damage-associated molecular patterns, such as high-mobility group box 1 (HMGB1) and biglycan [1].

Toll-like receptors (TLRs) are a family of 13 transmembrane receptors that recognize danger signals from microorganisms or damaged tissues. The expression of TLRs is not confined to immune cells alone. In kidneys, TLR1, 2, 3, 4, 6, and 9 are constitutively expressed in tubular epithelial cells, whereas TLR2 and 4 are normally present in endothelial cells [2,3,4]. Of these, TLR1, 2, 4, and 6 are present on the cell surface, while TLR3 and 9 reside on the intracellular endosome.

Several studies on both animals and humans provide compelling evidence that TLRs play key roles in renal IRI. Their expression in mouse tubular epithelial cells is increased following IRI, which is the initial biological response linked to the subsequent damage process [5]. The binding of TLR with danger signals prompts a downstream signaling cascade that results in the production of proinflammatory cytokines and chemokines, which then promotes the influx of leukocytes in the post-ischemic tissues. In knockout mouse models with genetic depletion of TLR2, 3, 4, and 9, the kidneys were more protected from IRI as demonstrated by diminished tubular injury and lower inflammatory cell infiltration than those in wild-type mice [4,6,7,8,9]. In humans, donor kidneys expressing mutated TLR4 (weaker affinity for HMGB1) had fewer inflammatory cytokines and a lower rate of delayed graft function than those expressing wild-type TLR4 [10].

However, the persistent absence of TLRs, as shown in an experimental model of stroke, may lead to delayed exacerbation of ischemic injury despite the reduced initial injury [11]. Thus, a temporary pharmacologic intervention to block TLRs during the acute phase of IRI may be a clinically relevant and desirable approach. Apart from effects on the kidneys, several studies have found that pharmacologic inhibition of TLR2 or TLR4 at the initial stage of injury reduces infarct size in experimental murine models of myocardial infarction and stroke [12,13,14,15].

**Figure 1:**
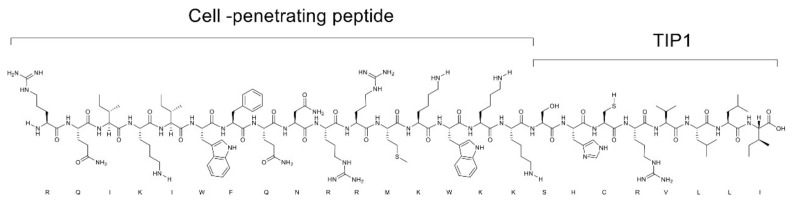
Chemical structure of Toll-like receptor-inhibitory peptide 1. Single-letter amino acid code is shown below the chemical structure.

We designed TLR-inhibitory peptide 1 (TIP1; Peptron Inc., Daejeon, Korea) by deriving the chemical structure from the Toll/interleukin 1 receptor (TIR) domain of myeloid differentiation primary response gene 88 (MyD88) adaptor-like (MAL). The molecular nature of TIP1 enables binding to the TIR domain of TLRs, thereby hindering the assembly of TLRs with their corresponding adaptor proteins and resulting in inhibition of TLR-mediated downstream signaling. For the effective delivery of TIP1 to the required site of action, we conjugated TIP1 to a cell-penetrating peptide (CPP), making a peptide of 24 amino acids with a formula weight of 3168 g/mol (Figure 1). Previously, we demonstrated that CPP-linked TIP1 (hereafter referred to as TIP1) blocked signal transduction of multiple TLRs, with the strongest effect on TLR4 [16]. In addition, this biological action was shown to lead to attenuated tissue damage in animal models of sepsis and inflammatory arthritis.

There have been continuous efforts to develop a treatment that can target the fundamental basis of renal IRI and improve its course. Despite these attempts, there are no compounds currently available for clinical use in renal IRI. Thus, with the goal of pursuing potential therapeutic candidates, we investigated the effect of TIP1 on a mouse model of bilateral renal IRI in which TLR signaling pathways are upregulated.

## 2. Results

### 2.1. TIP1 Administration Prior to IRI Alleviated Acute Kidney Injury

**Figure 2:**
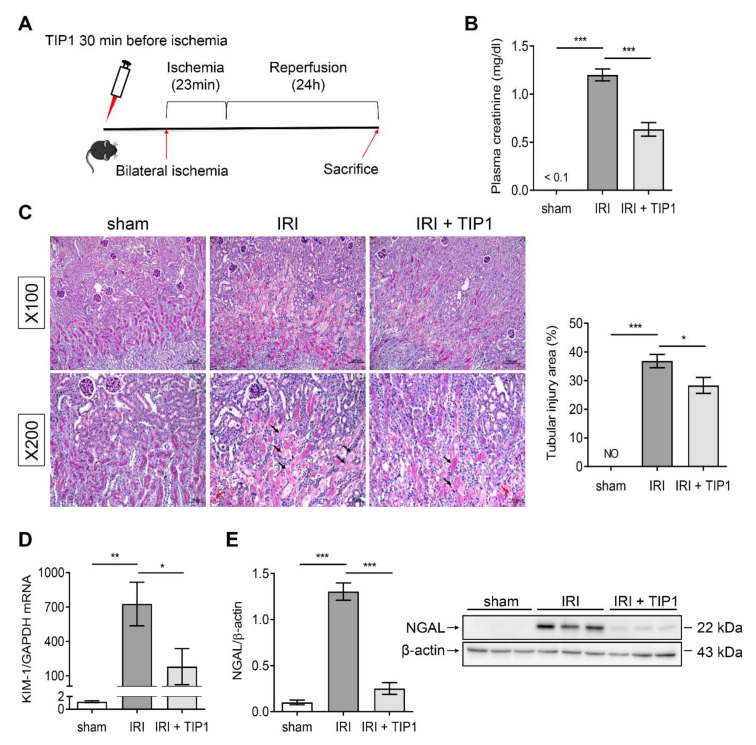
TLR-inhibitory peptide 1 (TIP1) attenuates kidney ischemia-reperfusion injury (IRI). (**A**) Experimental design. (**B**) Plasma creatinine levels. (**C**) The percentage of injured tubules in the deep cortex and the corticomedullary junction was assessed on periodic acid–Schiff staining. The lower images show higher magnification of the upper images. Black arrows indicate necrotic tubules with loss of brush border and red arrows indicate tubular casts. Asterisks represent dilated tubules. Scale bar = 100 (upper images) and 50 (lower images) μm. (**D**) KIM-1 mRNA expression in the kidney tissue. (**E**) Western blot analysis of NGAL in the kidney tissue. Sham (*n* = 6), IRI (*n* = 6), and IRI-TIP1 (*n* = 6). β-actin from the same membrane was used for densitometry. Data are expressed as mean ± SEM. * *p* < 0.05, ** *p* < 0.01, *** *p* < 0.001. Not observed (NO); kidney injury molecule-1 (KIM-1); neutrophil gelatinase-associated lipocalin (NGAL).

**Figure 3:**
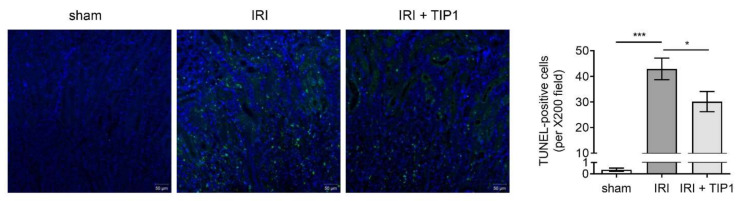
Protective effects of TIP1 against apoptosis in kidney ischemia-reperfusion injury. TUNEL staining and quantification of the number of TUNEL-positive cells in the harvested kidneys 24 h after IRI. Nuclei were counterstained with DAPI (blue). Data are expressed as mean ± SEM. Sham (*n* = 6), IRI (*n* = 6), and IRI-TIP1 (*n* = 6). * *p* < 0.05, *** *p* < 0.001. Scale bar = 50 μm. TLR-inhibitory peptide 1 (TIP1); terminal deoxynucleotidyl transferase-mediated deoxyuridine triphosphate nick-end labeling (TUNEL); 4′,6-diamidino-2-phenylindole (DAPI).

Renal IRI was induced by 23-min renal pedicle clamping after intraperitoneal vehicle or TIP1 injection (Figure 2A). The mice subjected to IRI showed an increase in plasma creatinine levels compared with that in the sham mice; however, TIP1 treatment before ischemic injury reduced the elevated creatinine concentration (Figure 2B). After 24 h of reperfusion, IRI caused widespread tubular cell necrosis and tubular dilation, predominantly involving the deep cortex and corticomedullary junction. This tubular damage was less extensive in mice pretreated with TIP1 (Figure 2C). Consistent with these findings, levels of neutrophil gelatinase-associated lipocalin (NGAL) protein and kidney injury molecule-1 (KIM-1) mRNA increased to a much lesser extent in TIP1-treated mice than those in untreated mice (Figure 2D,E). Terminal deoxynucleotidyl transferase-mediated deoxyuridine triphosphate nick-end labeling (TUNEL) staining also showed that TIP1 pretreatment significantly reduced the number of apoptotic cells induced by IRI (Figure 3).

### 2.2. TIP1 Reduced Cytokine and Chemokine Expression Following IRI

**Figure 4:**
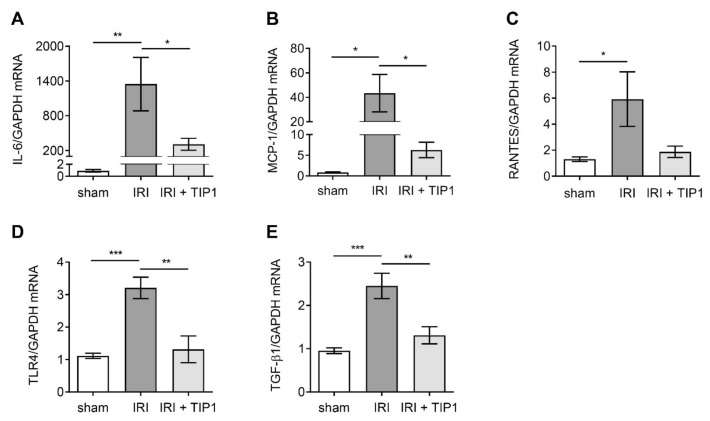
Inhibitory effect of TIP1 on proinflammatory cytokine and chemokine following kidney ischemia-reperfusion injury. mRNA expression of IL-6 (**A**), MCP-1 (**B**), RANTES (**C**), TLR4 (**D**), and TGF-β1 (**E**) in kidney tissue. Kidney tissues were harvested from sham (*n* = 6), IRI (*n* = 6), and IRI-TIP1 (*n* = 6) groups 24 h after IRI. Data are expressed as mean ± SEM. * *p* < 0.05, ** *p* < 0.01, and *** *p* < 0.001. Interleukin-6 (IL-6); monocyte chemoattractant protein-1 (MCP-1); regulated on activation, normal-T-cell-expressed and -secreted (RANTES); Toll-like receptor 4 (TLR4); transforming growth factor-β1 (TGF-β1).

As the final consequence of TLR stimulation is the production of inflammatory cytokines, we quantified the mRNA levels of inflammatory cytokines to examine if TIP1 effectively blocks the TLR signaling pathway. The renal mRNA profiles of interleukin-6 (IL-6), regulated on activation, normal-T-cell-expressed and -secreted (RANTES), and monocyte chemoattractant protein-1 (MCP-1) increased in mice subjected to IRI, but those increments were relatively less in the TIP1 pretreatment group (Figure 4A–C). Similarly, the expression levels of TLR4 and TGF-β mRNA were upregulated by IRI; however, TIP1 administration prior to IRI lowered the magnitude of these mRNA increases (Figure 4D,E).

### 2.3. The Effect of TIP1 on Immune Cell Infiltration after IRI

**Figure 5:**
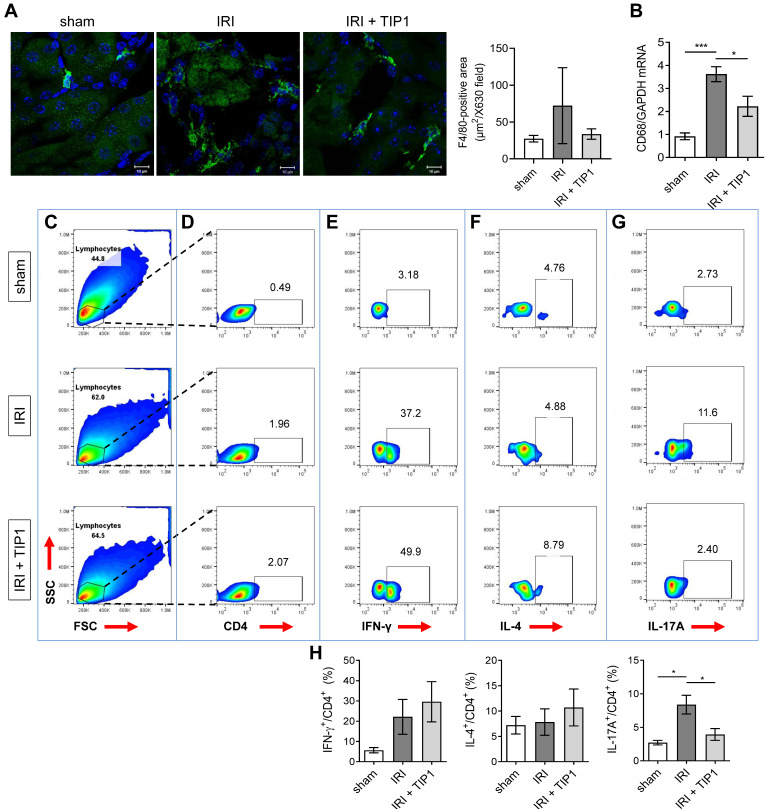
Inhibitory effect of TIP1 on immune cells following kidney ischemia-reperfusion injury. (**A**) Immunofluorescence staining and quantification of F4/80^+^ macrophages in the kidneys. Kidneys from sham (*n* = 4), IRI (*n* = 6), and IRI-TIP1 (*n* = 6) groups were harvested 24 h after IRI. Nuclei were counterstained with DAPI (blue). Scale bar = 10 μm. (**B**) CD68 mRNA expression in kidney tissue from sham (*n* = 6), IRI (*n* = 6), and IRI-TIP1 (*n* = 6) groups. The intrarenal lymphocyte (**C**) and CD4^+^ cell populations (**D**) at 24 h after IRI. The intrarenal IFN-γ^+^/CD4^+^ (**E**), IL-4^+^/CD4^+^ (**F**), and IL-17A^+^/CD4^+^ (**G**) cell populations at 24 h after IRI. (**H**) The percentage of intrarenal IFN-γ^+^, IL-4^+^, and IL-17A^+^ cell populations among CD4^+^ T cells. Kidneys from sham (*n* = 4), IRI (*n* = 6), and IRI-TIP1 (*n* = 6) groups were harvested at 24 h after IRI. Data are pooled from two independent experiments and expressed as mean ± SEM. * *p* < 0.05, *** *p* < 0.001. TLR-inhibitory peptide 1 (TIP1); forward scatter (FSC); side scatter (SSC); interferon-gamma (IFN-γ); interleukin-4 (IL-4); interleukin-17A (IL-17A); 4′,6-diamidino-2-phenylindole (DAPI).

In the immunofluorescence study, the F4/80^+^ staining area was increased after 24 h of reperfusion in comparison with sham-treated mice; however, this increase was less prominent in the TIP1-treated mice, although this pattern was not statistically significant (Figure 5A). Consistent with this pattern, IRI increased CD68 (macrophage marker) mRNA expression, and this upregulation was lowered by TIP1 pretreatment (Figure 5B).

Next, we performed flow cytometry to investigate whether the beneficial effects of TIP1 on innate immune cells and proinflammatory cytokines affect T cell population in injured kidneys. At 24 h after reperfusion, the intrarenal lymphocyte populations increased regardless of TIP1 treatment (Figure 5C). These lymphocytes were gated to CD4^+^ T cells (Figure 5D), among which the staining intensity for interferon-gamma (IFN-γ), IL-4, and IL-17A was examined (Figure 5E–G). This analysis revealed that the percentage of IL-17A-producing CD4^+^ T cells, indicative of T helper 17 (Th17) cells, increased 24 h after IRI, whereas this fraction had decreased in TIP1-pretreated group (Figure 5H). In contrast, the percentage of IFN-γ-producing CD4^+^ T cells (Th1 cells) and IL4-producing CD4^+^ T cells (Th2 cells) out of CD4^+^ T cells was not significantly changed after TIP1 administration.

### 2.4. The Protective Effect of TIP1 Was Associated with Reduced ROS 

**Figure 6:**
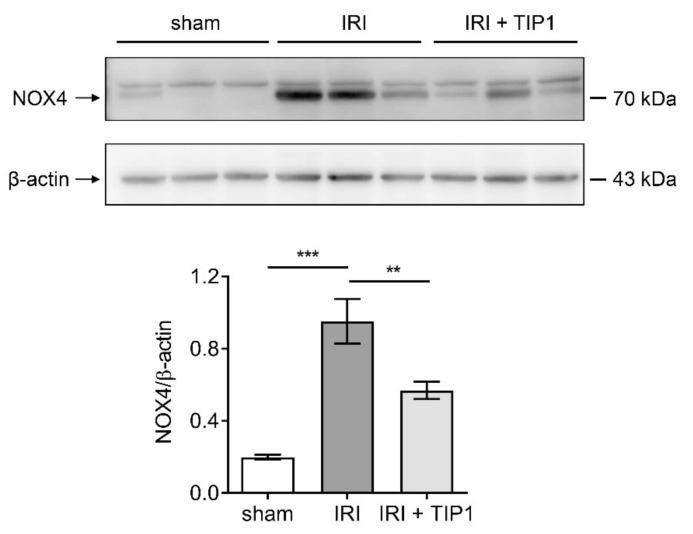
Inhibitory effects of TIP1 on oxidative stress in a renal ischemia-reperfusion model. NOX4 levels were quantified by western blot in kidney tissues from sham (*n* = 6), IRI (*n* = 6), and IRI-TIP1 (*n* = 6) groups. β-actin from the same membrane was used for densitometry. Data are expressed as mean ± SEM. ** *p* < 0.01, *** *p* < 0.001. TLR-inhibitory peptide 1 (TIP1); NADPH oxidases 4 (NOX4).

IRI is associated with increased ROS production in damaged tissues. Western blot analysis revealed that TIP1 pretreatment substantially attenuated the renal expression of NADPH oxidase 4 (NOX4) induced by IRI (Figure 6), suggesting that the renoprotective effect of TIP1 is associated with the reduction in ROS.

## 3. Discussion

This study showed the renoprotective effects of TIP1 against IRI, as demonstrated by improved tubular injury, reduced apoptosis, decreased levels of inflammatory cytokines, and oxidative stress. In addition, TIP1-pretreated reperfused kidneys displayed a tendency for lower macrophage infiltration and a decrease in the proportion of Th17 cells in CD4^+^ T cells. Our results provide further evidence that TLR plays a crucial role in the pathogenesis of renal IRI. In particular, we successfully demonstrate that pharmacological targeting of the TLR signaling pathway may be an effective strategy to attenuate the damage in ischemic acute kidney injury.

The TIR domain is a conserved region among all members of the TLR family and their associated adaptor proteins discovered to date. TIP1 interacts with the TIR domain of TLRs, where adaptor proteins, such as MyD88, MAL, TIR-domain-containing adaptor-inducing IFN-β (TRIF), and TRIF-related adaptor molecule (TRAM), are engaged through their TIR domains for downstream signal transduction. This mechanism of action enables TIP1 to block both MyD88- and TRIF-dependent pathways in multiple TLRs. In particular, TIP1 shows the most potent inhibitory action on TLR4, whose action plays a crucial role in endothelial, tubular epithelial, and immune cells in a different manner during IRI. A study using endothelial cells isolated from a mouse model of IRI revealed that TLR4 is involved in the expression of adhesion molecules in endothelial cells, whose TLR4 expression increases a few hours after reperfusion [3]. Following endothelial TLR4 expression, tubular cells predominantly express TLR4 in injured kidneys and produce proinflammatory cytokines. Since these cellular events eventually cause immune cell infiltration, TIP1 may indirectly influence macrophage and Th17 cell infiltration by reducing the expression of adhesion molecules and proinflammatory cytokines. In addition, TIP1 may affect immune cells directly as TLRs are expressed on both innate and adaptive immune cells, such as dendritic cells, macrophages, and T cells. 

CD4+ T cell differentiation is largely influenced by cytokine signals prevalent in the tissue microenvironment. In particular, a combination of TGF-β and IL-6 influences CD4+ T cells to polarize Th17 cells. Thus, TIP1 may tip the balance of CD4+ T cell subsets away from differentiation into Th17 cells by suppressing TGF-β and IL-6 expression in injured kidneys. Although the role of Th17 cells in ischemic acute kidney injury is not clearly defined yet, in vitro studies have revealed that Th17 cells exert proinflammatory action by chemoattracting neutrophils [17] as well as by influencing macrophages and tubular epithelial cells to produce inflammatory cytokines via IL-17 [18,19].

As a major source of ROS in the kidneys, NOX4 is abundantly present in proximal tubular cells, which are the most vulnerable to ischemic injury [20]. In the course of IRI, NOX4 expression is increased in injured proximal tubular cells and is considered a key culprit of ROS production in the phase of oxygen reintroduction [21]. Thus, a reduced increase in NOX4 levels via TIP1 action is expected to generate less oxidative stress in post-ischemic kidneys. In particular, Mkaddem et al. showed that a 28-kDa splice variant of NOX4 (although this was not measured in this study) interacts with TLR4 and is thereby involved in ROS production and apoptosis in posthypoxic tubular epithelial cells [22].

A persistent absence of TLRs may result in unwanted outcomes, as demonstrated by delayed exacerbation of an ischemic stroke despite reduced initial injury in TLR2 knockout mice [11]. Thus, a temporary pharmacologic intervention to block TLRs during the acute phase of IRI would be an appropriate approach. Several compounds, such as eritoran, TAK-242, OPN301, melatonin, pituitary adenylate cyclase-activating polypeptide 38, and ODN2088-encapsulated nanoparticles, have been found to ameliorate renal IRI through TLR inhibition; however, none of these have been approved for kidney injury encountered in clinical practice [23,24,25,26,27,28]. In contrast to eritoran and TAK-242, which have selective specificity for TLR4, OPN301 for TLR2, and ODN2088-encapsulated nanoparticles for TLR9, we found that, in our prior in vitro study using immune cells, TIP1 exerts an inhibitory action on TLR1 through TLR9, with the exception of TLR5. Since double knockout of TLR2 and 4 did not provide additional benefits against renal IRI compared with deletion of either TLR2 or TLR4 alone [29], the renoprotective effect of TIP1 could be derived from the predominant effect on TLR4. Or our result may reflect the net effect of TIP1 on multiple TLRs since other TLRs, such as TLR3 and 9, have been reported to mediate injury in kidneys subject to IRI [8,9].

Our results should be interpreted with caution as we did not investigate the long-term effects of TIP1 on post-ischemic kidneys. Although the pharmacologic action of TIP1 was considered short-lived during the initial phase of IRI, it is unknown whether pretreatment with TIP1 may hamper the regeneration of injured tubules despite reduced damage during the early period of reperfusion. Another limitation is that our experimental design of TIP1 pretreatment does not reflect common clinical settings, in which acute kidney injury occurs unpredictably and, therefore, treatment begins after injury has occurred. However, our approach can provide translational implications for certain situations in which cessation of renal blood supply is anticipated, such as major cardiac and vascular surgery and kidney transplantation. In addition, we did not examine the effects of TIP1 at the cellular level using tubular, endothelial, and immune cells. Lastly, we did not investigate whether TIP1 itself is harmful to the kidneys. Nonetheless, the amount of TIP1 administered was within the range in which cytotoxic effects were not observed in either HEK-Blue hTLR4 or RAW 264.7 cell lines [16].

Currently, there is no specific treatment for IRI, and little has been achieved with respect to its management despite better understanding of the underlying molecular processes. Our study demonstrated that TIP1 improved ischemic kidney injury and suggested a potential candidate that may be useful in clinical conditions in which IRI is involved.

## 4. Materials and Methods

### 4.1. Animal Study

Eight-week-old male C57BL/6 mice weighing 20–23 g (Dae Han Bio Link Co., Ltd., Chungcheongbuk-Do, Korea) were randomly divided into three groups: (1) sham: control mice in which only a flank incision was performed (2) IRI: the mice underwent bilateral renal pedicle clamping after vehicle injection, and (3) IRI-TIP1: the mice underwent bilateral renal ischemia after the administration of an intraperitoneal TIP1 administration. Briefly, 30 min after an intraperitoneal injection of TIP1 (10 nmol/g) or 0.9% normal saline, the mice were anesthetized using Rompun (10 mg/kg; Bayer, Leuverkusen, Germany) and Zoletil (30 mg/kg; Virbac Laboratories, Carros, France). The kidney pedicles were then exposed on both sides through a bilateral flank incision and clipped for 23 min using microaneurysm clamps [30]. Mice were placed on a heating pad at 38–39 °C in a supine position to maintain body temperature during the surgical procedure. Mice were euthanized after 24 h of reperfusion with intraperitoneal injection of Rompun and Zoletil; blood was collected, and kidneys were harvested. Plasma creatinine levels were determined using the VetTest 8008 (IDEXX Laboratories, Westbrook, ME, USA). All animal experiments were performed in compliance with the guidelines of the Animal Research Ethics Committee of Kyung Hee University and approved by the Institutional Animal Care and Use Committee of Kyung Hee University Hospital at Gangdong (KHNMC AP 2019–002; approval date: 22 February 2019).

### 4.2. Histological Analysis

For morphological assessment, formalin-fixed and paraffin-embedded sections (4 µm) were stained with periodic acid–Schiff. Tubular injury was assessed on the basis of morphological changes, including tubular dilation, tubular cell necrosis, cast formation, and loss of brush border, by two independent researchers blinded to the experimental groups. The parenchyma in the deep cortex and the corticomedullary junction were examined in their entirety in all microscopic fields covering the entire slide to obtain the percentage of injured area.

### 4.3. Western Blot

Protein samples were extracted from the homogenized kidneys using the PRO−PREP protein extraction solution (iNtRON Biotechnology, Seongnam, Korea). After the measurement of protein concentration by the BCA Protein Assay Kit (Thermo Fisher Scientific, Waltham, MA, USA), 30 µg of protein was loaded on an 8–15% SDS-PAGE gel and were transferred onto a polyvinylidene difluoride membrane (Millipore, Madrid, Spain) by electroblotting. The membrane was blocked for 1 h using 5% skim milk and incubated overnight at 4 °C with primary antibodies against NGAL (1:1000; R & D System Inc., Minneapolis, MN, USA), NOX4 (1:1000; Santa Cruz Biotechnology, Santa Cruz, CA, USA), and β-actin (1:5000; Santa Cruz Biotechnology). The next day, the membranes were incubated for 2 h at room temperature with secondary anti-rabbit or anti-goat antibodies (1:10,000) linked to horseradish peroxidase. Immunoreactive bands were detected by enhanced chemiluminescence. β-actin was used as an internal control.

### 4.4. Quantitative Real-Time PCR

RNA was extracted from the kidney tissue using the Total RNA Isolation Kit (Macherey-Nagel, Düren, Germany) and was reverse-transcribed into complementary DNA (cDNA) using random primers (Promega, Madison, WI, USA), deoxynucleotide mix (Takara Bio Inc., Shiga, Japan), and Maloney-murine leukemia virus reverse transcriptase (Mbiotech Inc., Gyeonggi, Korea). Thereafter, real-time PCR was run on the Applied Biosystems^®^ StepOnePlus™ System with a final volume of 20 µL containing 1 µL of cDNA, 5–10 pmol of each of the sense and antisense primers, 10 µL of Power SYBR Green Master Mix (Applied Biosystems, Foster City, CA, USA), and nuclease-free water. The primer sequences used are shown in Table 1. Each sample was run in duplicate, and data were analyzed using the ∆∆CT method with normalization to GAPDH expression.

### 4.5. Immunostaining

Kidneys were embedded in optimal cutting temperature compound (Sakura Finetec, Tokyo, Japan) and cut into 6-μm-thick sections using a cryostat (Leica Biosystems, Wetzlar, Germany). Slides were blocked for 1 h at room temperature with 1% bovine serum albumin in phosphate-buffered saline (PBS). Then, the kidney sections were incubated overnight at 4 °C with a primary antibody against F4/80 (1:50; Bio-Rad, Hercules, CA, USA). After washing with PBS, the sections were incubated for 1 h at room temperature with a secondary antibody, anti-rat Alexa Fluor 488 (1:200; Invitrogen, Carlsbad, CA, USA), and were then counterstained with DAPI (Invitrogen) for 30 min at room temperature. F4/80 staining was quantified in 7–10 randomly chosen ×630 microscopic images obtained from the deep cortex and corticomedullary regions, and the results were expressed as F4/80-positive area per ×630 field.

TUNEL was performed to assess apoptosis using an in-situ cell death detection kit (Roche Applied Science, Indianapolis, IN, USA) according to the manufacturer′s protocol. Apoptotic cells were counted from 7 to 10 randomly chosen ×200 microscopic images of the deep cortex and corticomedullary areas. Images of F4/80 staining and TUNEL were captured using a Zeiss LSM 700 confocal microscope (Carl Zeiss, Oberkochen, Germany) and analyzed using ImageJ software.

### 4.6. Flow Cytometry

Kidneys were harvested after perfusion and disrupted mechanically using a Stomacher 80 Biomaster (Sewart, Worthing, UK). Single-cell suspensions were prepared by passing the homogenates through a 100-μm cell strainer. After centrifugation, the cell pellet was resuspended in 40% Percoll. Then, the samples were layered on top of 80% Percoll and centrifuged at 2000× *g* for 30 min. The cell layer located in the middle of the tube was collected and RBCs were removed using RBC lysis buffer (Sigma-Aldrich, St. Louis, MO, USA). Isolated cells were filtered through a 70-μm cell strainer and then stimulated with a cell stimulation cocktail (PMA, Ionomycin, GolgiStop) for 2 h at 37 °C in RPMI-1640 media. Thereafter, the cells were fixed, permeabilized, and stained with antibodies against CD4 (BioLegend, San Diego, CA, USA), IFN-γ (BD Biosciences, Bedford, MA, USA), IL-4 (BD Biosciences), and IL-17A (BioLegend). Fluorescence signals were detected using a FACS Calibur instrument, and the cell frequencies were analyzed using CellQuest (BD Biosciences) and FlowJo software (FlowJo LLC, Ashland, OR, USA).

### 4.7. Statistical Analyses

All values are expressed as mean ± standard error of the mean. Data were analyzed with ANOVA with Tukey post hoc analysi*s* (if the number of mice was the same among groups) or Bonferroni correction (if the number of mice was different among groups) using SPSS software, version 22 (SPSS, Chicago, IL, USA). A value of *p* < 0.05 was considered statistically significant.

## 5. Conclusions

The introduction of a pharmacologic agent to prevent or dampen renal IRI has remained an unmet need for several decades. TLRs are the forefront sensor for danger signals induced by IRI, and their activation results in the production of proinflammatory cytokines and chemokines. Our study applied a multiple-TLR-blocking agent, TIP1, to a mouse model of bilateral renal IRI, and highlighted that inhibition of the TLR signaling pathway during the initial stage of IRI resulted in attenuated kidney damage accompanied by decreased levels of apoptotic cells and oxidative stress and decreased inflammatory cell infiltration. This finding suggests that TIP1 may offer a potential renoprotective effect in ischemic kidney injury.

## Figures and Tables

**Table 1:** Primer sequences used for real-time PCR assays.

Gene	Primers
KIM-1	5′-TCTATGTTGGCATCTGCATCG-3′
	5′-GAAGGCAACCACGCTTAGAGA-3′
IL-6	5′-GACTGATGCTGGTGACAA-3′
	5′-GTGAAGTGGTATAGACAGGTC-3′
MCP-1	5′-CATCCACGTGTTGGCTCA-3′
	5′-AACTACAGCTTCTTTGGGACA-3′
RANTES	5′-GCTCCAATCTTGCAGTCGT-3′
	5′-CCTCTATCCTAGCTCATCTCCA-3′
TLR4	5′-ATGGCACTGTTCTTCTCCTG-3′
	5′-AGCTCAGATCTATGTTCTTGGTTG-3′
TGF-β1	5′-CAACAATTCCTGGCGTTACCTTGG-3′
	5′-GAAAGCCCTGTATTCCGTCTCCTT-3′
CD68	5′-ACCGCCATGTAGTCCAGGTA-3′
	5′-ATCCCCACCTGTCTCTCTCA-3′
GAPDH	5′-TGTGTCCGTCGTGGATCTGA-3′
	5′-TTCGTGTTGAAGTCGCAGGAG-3′

kidney injury molecule-1 (KIM-1); interleukin-6 (IL-6); monocyte chemoattractant protein-1 (MCP-1); regulated on activation, normal T cell expressed and secreted (RANTES); Toll-like receptor 4 (TLR4); transforming growth factor-β1 (TGF-β1).

## Data Availability

Not applicable.
